# The Impact of Collaborative Learning and Personality on Satisfaction in Innovative Teaching Context

**DOI:** 10.3389/fpsyg.2021.713497

**Published:** 2021-09-29

**Authors:** Fei-Fei Cheng, Chin-Shan Wu, Po-Cheng Su

**Affiliations:** ^1^Institute of Technology Management, National Chung Hsing University, Taichung City, Taiwan; ^2^Department of Information Management, Tunghai University, Taichung City, Taiwan

**Keywords:** flipped education, need for cognition, learning self-efficacy, collaborative learning, learning satisfaction

## Abstract

Flipped teaching is one of the most popular innovative teaching methods which has attracted a lot of attention and lead to amount of discussion in recent years. Many educators have generally encountered same doubt when implementing flipped education: Is this kind of teaching mode only applicable to students with high learning achievements? In addition, collaborative learning is often applied in flip teaching and it is also an issue worth to explore. In this study, both quantitative and qualitative studies were conducted to examine the potential factors in affecting the learners’ satisfaction in flipped education. The survey results from 171 participants showed that collaborative learning and need for cognition are significant predictors of learning satisfaction. In addition, a deeper look at the collaborative learning process was further examined by conducting deep interview. A total of 12 students from 6 different flipped-teaching courses participated the interview. The findings suggested that arranging some activities to encourage students to know each other before class that helps students find corresponding group and facilitates their expertise for collaborative learning. The mechanism significantly influenced team members’ engagement, discussion atmosphere, and efficiency. In addition, when learning tasks diversity, it will also enhance students’ innovative ability, empathy, and even promote mutual learning.

## Introduction

In recent years, flipped teaching has attracted considerable attention and aroused widespread discussion. Since 2011, the search trend of relevant keywords on Google has increased exponentially (Abeysekera and Dawson, 2015). Many studies have compared student performance before and after the implementation of flipped teaching, evidencing that flipped teaching can help improve academic performance (O'flaherty and Phillips, 2015). When the science control system of the Department of Mechanical Engineering of Seattle University implemented flipped teaching, the course divided students into two different groups: the traditional learning method group and the flipped learning method group. Results show that the group receiving flipped teaching generally performed better in tests and examinations and had a higher degree of mastery of design issues (Mason et al., 2013). A course on renal drug therapy conducted flipped teaching to evaluate its impact on students’ professional performance. The results show that compared with the previous year’s performance in a traditional classroom environment, students’ performance in the final exam improved significantly (Pierce and Fox, 2012). Most of the topics discussed in the existing literature mainly focus on the comparison of the effectiveness and acceptance of flipped teaching and traditional teaching among students.

After a systematic review of research related to flipped teaching, O'flaherty and Phillips (2015) pointed out that individual differences can be explored in the future, for example, whether there are specific demographics or personalities that can predict students’ responses to flipped lessons. To explore the individual differences of students under flipped teaching, this study refers to the research of Abeysekera and Dawson (2015). Although the research on flipped teaching has been conducted in a variety of domains (Chang and Hwang, 2018; Lee and Wallace, 2018; Javier et al., 2020), this study aimed at examining the potential factors in affecting the learning satisfaction in flipped education by combining both qualitative and quantitative study. Thus, the first objective of this study is to examine the influential factors of learners’ satisfaction from the perspective of personality, self-efficacy, and collaborative learning. Specifically, this study focuses on the impact of students’ personality traits and collaborative learning on learning satisfaction under flipped teaching so as to understand the response of individual differences to flipped teaching. This study uses the cognitive needs theory and learning self-efficacy as the entry point to explore personality traits and provides a reference for educators who plan to practice in flipped teaching in the future.

Further, as most of the flipped teaching courses require students form into groups and collaborative with each other to finish the projects, the second objective of this study is to look deeper into the collaborative learning process for students participating the flipped learning. Thus, deep interviews were conducted to understand the collaborative learning process when the students participated the flipped learning and the findings can provide significant insight for educators who want to teach in a more innovative way and increase students’ engagement in flipped teaching.

## Literature Review

### Flipped Teaching

The term “flipped teaching” is commonly used to describe a teaching method wherein the completion of homework after class is carried out in the classroom and the classwork is to be completed by the students themselves before class (Abeysekera and Dawson, 2015). The idea of flipped teaching first occurred as an accidental discovery by a high school chemistry teacher in the United States when he wanted to conduct remedial classes for absent students. He bought a set of software and uploaded the classwork teachings on the Internet so that absent students could keep up with their studies. However, in addition to the students who were absent from class, students who had originally attended the class also used the online teaching resources to review the course content and benefited from it. This discovery made Bergmann and Sams rethink the allocation of class time in the teaching process (Tucker, 2012).

Many studies have proven that the flipped teaching method can improve students’ learning motivation. In a statistics course at a university, it is understood through interviews that students are more willing to accept collaborative learning and innovative teaching methods than traditional teaching (Strayer, 2012). After the introduction of flipped teaching in the British chemistry curriculum, students expressed that they preferred this interactive mode, because it gave them more opportunities to develop more advanced learning skills in the classroom than before (Yeung, 2014). It not only improves learning motivation but also stimulates students’ active learning because of the changes in the teaching process. In addition to the teaching content of the course itself, when the course is conducted in the form of group discussions, communication and critical thinking abilities also improve. In a study on the implementation of flipped teaching in nursing courses, students had more opportunities to discuss and solve unfamiliar problems with their peers and teachers in the classroom. Through the redesigned curriculum, students were required to criticize various scenarios, collect information, and provide insights for patients. Such learning activities combine knowledge of patient assessment, critical thinking, and evaluation skills (Ferreri and O’connor, 2013).

The European Higher Education Framework proposes a shift from the previous one-way teaching of courses by teachers to a student-centered learning approach (Schreurs and Dumbraveanu, 2014). Honeycutt and Garrett (2014) refer to the flipped classroom as paying attention to a learner’s learning status through their participation in solving problems, creating, criticizing, and integrating problems with peers and teachers in the classroom. Bergmann and Sams (2012) believe that the core of flipping is to focus on students’ needs, and Bloom Taxonomy provides a framework for judging whether it is flipped teaching: courses centered on past lectures usually focus on the lower level of Bloom Taxonomy, such as the cognition and understanding of basic knowledge. Teachers with flipped teaching will focus more on the high-level learning results of Bloom’s taxonomy in the classroom, such as analysis, judgment, and creation.

Sáiz Manzanares et al. (2017) analyze the effect of blend learning on students’ learning outcomes. The results showed that different learning patterns can predict student learning outcomes. Further, Yin and Yuan (2021) examined the learning performance in a blended learning environment in China and the factors of perceived precision teaching, self-efficacy, learning motivation, and social presence were examined. The results indicated that all the predictors showed significant effect on learning performance, of which self-efficacy is one of the most important factors in predicting learning performance. In addition, Yokoyama and Miwa (2021) examined the effects of self- and peer-assessment on the growth of learning goal orientation. Results from the experiment showed that peer-assessment is effective in enhancing the growth of learning goal orientation.

The above discussion revealed that studies on flipped teaching are varied. However, most of the studies are focusing on language learning. For example, Lee and Wallace (2018) provided empirical evidence about whether flipped learning can promote students’ English learning. Andujar et al. (2020) examine the effect of integrating the flipped teaching and the usage of mobile devices in language learning. Further, Amiryousefi (2019) investigated the effects of flipped learning on EFL (English as a foreign language) learners’ engagement.

This study refers to Abeysekera and Dawson (2015) to explore flipped teaching with the following three characteristics: (1) process-oriented and inquiry-based learning, (2) peer-based team learning, and (3) peer interaction and learning.

### Learning Satisfaction

Learning satisfaction has always been a very important research indicator in education-related research. According to a study by Ko and Chung (2014), there is a significant positive correlation between student learning satisfaction and academic performance. A report on innovative teaching also pointed out that students’ learning satisfaction directly affects their academic performance; thus, it is also one of the main items used to measure or predict learning effectiveness (Lee, 2011).

Chang and Chang (2012) defined learning satisfaction as the degree of happiness that students experience after learning activities. Learning satisfaction exists in the balance between personal expectations and self-realization. When the results of self-realization are equal to or higher than personal expectations, learning satisfaction can be improved; however, when the results of self-realization are not as good as personal expectations, one cannot obtain a sense of achievement in learning (Martin, 1988).

Many factors can affect students’ learning satisfaction. In a study on learning satisfaction among adults receiving computer-related skills teaching, divided learning satisfaction into five items: teacher’s teaching, classroom materials, learning outcomes, interpersonal relationships, and learning environment. In a survey of learning satisfaction among college students, the questionnaire was divided into five items: learning environment, academic performance, administrative services, interpersonal relationships, and attitudes toward teachers and administrators (Starr, 1971). Corts et al. (2000) used five environmental factors to study how student satisfaction was affected. The results of the study show that employability development and curriculum planning have the deepest impact on student satisfaction. Teven and Mccroskey (1997) also prove that teachers’ attention toward students’ learning conditions and their interactions with students contribute to improving students’ learning satisfaction.

Based on the above views of scholars, although the factors affecting learning satisfaction have different research results and opinions because of the research focus of scholars, in fact, the external factors that affect students’ learning satisfaction are mainly constituted by teachers’ teaching methods, arrangement of learning activities, curriculum content planning, classroom teaching materials, learning outcomes, employment skills training, interpersonal interactions with peers and teacher interactions, and other factors.

### Learning Self-Efficacy Theory

The self-efficacy theory was first proposed by Bandura in 1977, and Bandura defined it as the degree to which people believe they can accomplish tasks and achieve goals (Bandura, 2010). The influencing factors of self-efficacy mainly come from the following four types: (1) through the successful experience of learning to build a stronger self-efficacy; (2) by seeing people similar to themselves who have worked consistently to achieve success and thus believing that they have similar abilities to be successful; (3) through the verbal encouragement of others, which makes people believe that they have the relevant abilities needed to complete the task, and they are willing to try to improve their self-efficacy; and (4) physiological conditions, negative emotions, and unhealthy physical conditions will lead to low self-efficacy (Bandura, 2010).

Because self-efficacy affects people’s feelings, thinking, and behaviors, it has been widely studied and applied in many fields after it was proposed, which include addiction problems (Bandura, 1995), smoking behavior (Garcia et al., 1990), and athletic performance (Barling and Abel, 1983). In education research, the value of self-efficacy has drawn increasingly more attention (Pajares, 1997). In education, research on self-efficacy focuses on the following three aspects: (1) the relationship between self-efficacy and university majors and career choices (Lent and Hackett, 1987); (2) teachers’ self-efficacy beliefs, teaching practices, and student academic performance (Ashton and Webb, 1986); and (3) the relationship between students’ learning self-efficacy beliefs and academic achievement (Bartimote-Aufflick et al., 2016).

In the research on the relationship between students’ learning self-efficacy and academic performance, students with high learning self-efficacy treat it as a challenge when they encounter difficulties in learning. Such students set challenging goals and continue to work hard. When faced with failure, they attribute the failure to insufficient effort or insufficient knowledge and skills, and they are more willing to keep working hard. On the contrary, students with low self-efficacy choose to escape when faced with difficulties and do not ask for learning goals. They usually give up easily when faced with problems, because they regard insufficient learning self-efficacy as insufficient ability (Bandura, 2010).

According to Bandura’s narrative, it is reasonable to infer that learning self-efficacy has a positive effect on learning effectiveness. In many studies, learning self-efficacy has also been proven to be an important indicator used to predict academic performance (Elias and Loomis, 2002). In addition, because learning outcomes affect learning satisfaction (Starr, 1971), Aitken (1982) also pointed out that grade point average (GPA) has a positive effect on learning satisfaction. After reviewing the literature related to both learning self-efficacy and learning satisfaction, the following hypothesis was proposed:

*H1*: Learning self-efficacy will positively influence learning satisfaction.

### Need for Cognition

Need for cognition is a personality trait. Cohen et al. (1955) first defined this concept as “a need for individuals to organize their experience meaningfully.” Cacioppo and Petty (1982) modified this viewpoint, thinking that cognitive needs reflect people’s enthusiasm for activities related to cognitive thinking types.

People with low cognitive needs do not like cognitive tasks when dealing with complex problems and tend to rely on others or even expert opinions (Petty et al., 1981). People with high cognitive needs are relatively more willing to devote themselves to thinking tasks or work and are more likely to use systematic thinking to process information. Such people are described as having a high degree of intrinsic motivation, aspiration, and curiosity, so they actively search for information (Olson et al., 1984). As for causes of individual differences in cognitive needs in various social environments, the main reason is intrinsic motivation. This individual difference is stable for a while and not easy to change (Cacioppo et al., 1996).

Most contemporary research involving the cognitive needs theory is based on the discourse of Cacioppo and Petty (1982). Research involving cognitive needs includes social cognitive psychology, medicine (Haugtvedt et al., 1992), and online consumer behavior (Lin et al., 2011). In the literature related to education, Sadowski and Gülgös (1996) studied how cognitive needs affect academic performance, and the results prove that students with high cognitive needs achieve more academic achievements than those with low cognitive needs, because the former can deal with information more effectively than the latter. Dole and Sinatra (1998) also put forward similar research viewpoints, because students with high cognitive needs also have a higher level of performance in speculation and problem-solving during the learning process; on the contrary, students with lower cognitive needs have a lower level of performance. In a study that discussed the relationship between cognitive needs and the ability to solving complex problems (Nair and Ramnarayan, 2000), it was pointed out that current cognitive needs have a significant positive correlation with solving complex problems, because people with high cognitive needs collect information and make multifaceted decisions about problems; they are more likely to succeed in solving problems.

According to the above discussion about cognitive needs in education literature, we understand that cognitive needs have a significant positive correlation with learning effectiveness. In a study by Elias and Loomis (2002), the correlation between cognitive needs, learning self-efficacy, and learning effectiveness was verified. It has been proven that cognitive needs and learning self-efficacy are important predictors of GPA (Strobel et al., 2019). Besides, it was also found that the relationship between cognitive needs and GPA was affected by the mediation of learning self-efficacy. Thus, the following hypotheses were proposed:

*H2*: Need for cognition will positively influence learning satisfaction.

*H3*: Need for cognition will positively influence learning self-efficacy.

### Level of Collaborative Learning

Collaborative learning is defined as when students achieve a common learning goal, they complete it in a group and are responsible for each other’s learning (Gokhale, 1995). It is worth noting the difference between “cooperative learning” and “collaborative learning.” Cooperative learning refers to a model in which a learning task is divided into subtasks that can be solved independently by partners at the beginning. Collaborative learning is solving a problem together in an asynchronous and interactive way. The difference between the two is that collaborative learning emphasizes the discussion in the process of participating in tasks and believes that cognition must be adjusted through communication between students (Curtis and Lawson, 2001). Verdejo (1996) emphasizes collaborative learning based on dialogue.

Gokhale (1995) pointed out that active exchange of ideas within the group will not only increase students’ interest but also promote critical thinking. Studies have shown that compared to individual learning, collaborative learning provides students the opportunity to discuss and have a higher level of thinking, and information can also be memorized for longer.

According to the observation of Tuckman and Jensen (1977), it is pointed out that the interpersonal relationship between group members in collaborative learning will generally go through the following four stages: (1) Formation stage: a transitional period when group members are not familiar with each other; (2) Conflict stage: the transition period in the growth of the group, when group members adapt to each other and run-in; (3) Cohesion stage: If the conflict is handled properly, a balance that is acceptable to the members of a group is sought, gradually forming a consensus, and the cohesion of the group will increase day by day; and (4) Execution stage: team members will focus on the completion of the task and the achievement of the goal. Members depend more on each other, and each person’s role positioning will be more productive.

In the collaborative learning environment, regardless of the level of learning achievement, students generally perform better than their peers who study alone (Aitken, 1982), and in the process of collaborative learning, students’ communication with each other is also considered helpful (Bruffee, 1982). According to the current research on collaborative learning, it is clearly pointed out that collaborative learning can improve learning more effectively (Hertz-Lazarowitz et al., 2013) and reinforce students’ satisfaction with the entire learning process (Bligh, 1998; Ocker, 2001). Thus, the following hypothesis was proposed:

*H4*: Collaborative learning will positively influence learning satisfaction.

## Research Method

### Research Design

**Figure 1:**
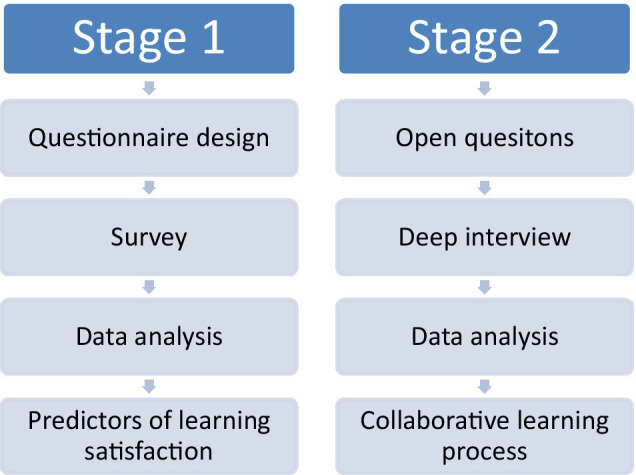
Two-staged research design.

The study applied both quantitative and qualitative research methods to examine the factors in flipped teaching outcome. In the first stage, a survey was conducted and questionnaires were distributed to students who experienced the flipped learning method. The objective of the survey is to examine predicting factors of learning satisfaction. The study uses need for cognition, learning self-efficacy, and collaborative learning as the predictors that affect the satisfaction of flipped teaching. In the second stage, deep interviews were conducted to understand the collaborative learning process as it is one of the most important mechanisms in flipped learning. The objective of the second stage is to explore the learners’ collaborative learning process in terms of team formation, discussion atmosphere, discussion efficiency, decision-making mode, cooperation mode, and cross-domain learning. In the second stage, semi-structured in-depth interviews were conducted. The interview comprises open questions, starting with the interviewee’s personal background, including questions about name, gender, school, department, grade, and major courses, and then cutting into the core questions of the research gradually. The two-staged research design is depicted in Figure 1.

### Data Collection

All the participants in this study (including stage 1 and stage 2) were recruited from six courses which were given as flipped teaching methods. The students were either asked to fill out the questionnaire for qualitative research or invited as an interviewee for qualitative research in this study.

This study refers to the flipped teaching model proposed by Abeysekera and Dawson (2015). The flipped teaching curriculum must have the following three elements: (1) process-oriented inquiry learning, (2) team-based learning, and (3) peer learning. According to this standard, a total of six courses have been selected as the experimental situation, which was described in Table 1.

**Table 1:** The description of courses selected in the study.

Course name	Course objective
Entrepreneurship Management	Students must work in groups of 5–6 people, start a company in the form of practical entrepreneurship, and learn related professional knowledge, such as business models and operation management through the process of selling goods or services
Marketing Management	The course cooperates with a public library in holding a marketing activity to promote reading. Students would provide marketing plans in a group of 5–6 during the participation process
Knowledge Creation and RandD Management	Students are required to complete an essay in a group of three people at the end of the term, and students must learn statistics and research methods during the implementation process
Introduction to Computer and Network Security	The course requires students to complete a project related to information security in the form of a group at the end of the semester
Reading Industry and Cultural Communication	The course requires students to actually establish a publishing house to publish a book at the end of the semester, in order to understand the actual operating conditions of the publishing industry through the process of participation
Entrepreneurship Fundraising Training Camp	The course requires students to actually put the business plan on the fundraising platform to raise funds and acquire relevant knowledge of plan writing, service design, and financial management in the process

The sample of survey and interview are students from the same pool (the six courses listed in Table 1). The objective is to examine the factors of learning satisfaction from qualitative study. At the same time, students from the same courses were invited to participate the interview in order to illustrate in more detail about the collaborative learning process in flipped classroom.

The data in this study were collected in two ways. First, students who take the courses illustrated in Table 1 were invited to fill out the questionnaire at the end of the semester. The questionnaires were distributed under the permission of the instructors, and a total of 171 valid samples were returned.

Second, the participants in qualitative study were invited from the six courses in Table 1. Student was randomly selected to represent each of the top 25% and bottom 25% of the course scores. A total of 12 students from the six courses participated in this research interview. To protect the privacy of interviewees, the student names on the recording form are presented anonymously.

### Research Framework

**Figure 2:**
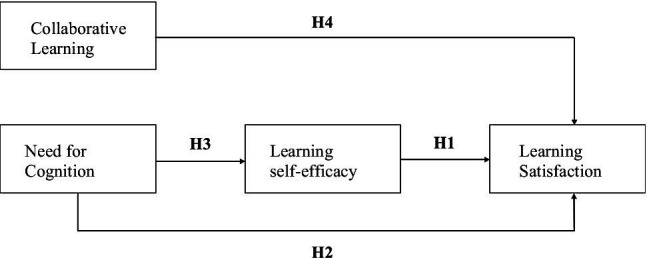
Research framework.

The research framework of this study is depicted in Figure 2, in which two independent variables (collaborative learning and need for cognition), one mediator (learning self-efficacy) and one dependent variable (learning satisfaction), were included.

## Research Analysis and Results

### Quantitative Results

SMART PLS (partial least square) software was used for data analysis, and structural equation model (SEM) was applied. Structural equation model is composed of two parts: measurement model and structural model. The measurement model is used to observe the relationship between potential variables; structural model is used to measure the relationship between variables and potential variables. This study applied confirmatory factors in the measurement model for theory verification and applied path analysis in the structural model to explore the causal relationship between variables.

#### Demographics

In terms of the gender distribution of the participants (as shown in Table 2), the proportion of males was 43.86% of the total subjects, and the proportion of females was 56.14%. In terms of grade distribution, 28.65% of juniors formed the group with the highest distribution, followed by 27.49% of seniors (and above), 26.90% of the first year of graduate school, 7.01% for both the freshman and sophomores, and lastly, 2.92% for the second year of graduate school (and above); as for the distribution of the colleges, the College of Management had the most students, accounting for 53.80% of the total, followed by 14.04% in the College of Electrical Engineering and Computer Science, 12.87% in the College of Agricultural and Natural Resources, 9.94% in the College of Liberal Arts, 4.09% in the College of Science, 2.34% in the College of Engineering, 1.75% in the College of Law and Politics, and 1.17% in the College of Life Sciences.

**Table 2:** Demographics (*N*=171).

Variable name	Variable category	*N*	%
Course	Entrepreneurial Management	85	49.71
	Marketing Management	38	22.22
	Knowledge Creation and RandD Management	13	7.60
	Introduction to Computer and Internet Security	20	11.7
	Reading Industry and Cultural Communication	13	7.60
	Entrepreneurial Bidding Training Camp	2	1.17
Gender	Male	75	43.86
	Female	96	56.14
Grade	Freshman	12	7.01
	Sophomore	12	7.01
	Junior	49	28.65
	Senior (and above)	47	27.49
	Graduate school	51	29.82
College	College of Liberal Arts	17	9.94
	College of Agricultural and Natural Resources	22	12.87
	College of Management	92	53.80
	College of Electrical Engineering and Computer Science	24	14.04
	Other	16	9.35

Since the students at the College of Management were set as the largest number of participants in this study, the demographic variables were also affected by the composition of the grade and the college of the testing class. First, gender was the most influential part, as most of the students in the College of Management were female, which led to the reason that most of the subjects were female.

In addition, in terms of grades, it is noteworthy that most departments and colleges generally require courses with a higher level of implementation, and they are generally offered in the upper grades of the university department. This also explains why the distribution of the test subjects was mainly junior and senior students and the first grade of graduates.

#### Reliability and Validity Analysis

Validity refers to the theoretical extent to which the questionnaire can measure. The commonly applied method to test the validity in structural equation model is the confirmatory factor analysis in the measurement model. In the same factor dimension, if the factor load of each topic is larger, it means the degree of convergence is greater. Usually, it must be greater than 0.7, and the average variance extracted (Schreurs and Dumbraveanu) must be greater than 0.5. Based on these data as the test standard, after running the PLS statistical analysis, the items that did not meet the factor load were deleted: The first 18 questions about cognitive needs retained the first 1, 8, 10, 11, 12, 14, and 15 questions; 10 questions about learning self-efficacy were reserved for Questions 3, 4, 7, 8, 9, and 10; for the level of collaborative learning, except for Question 6, the remaining 6 questions were reserved; for learning satisfaction, the questions were reserved except for Question 6. The AVE of the retained items after organizing all the dimensions was greater than 0.5.

Reliability represents the stability of the subject’s answer. The most applied verification method is Cronbach’s alpha. When the Cronbach’s alpha coefficient is greater than 0.7, the question items of the scale which the respondents fill in are consistent. The Cronbach’s alpha values of the above items that passed the validity test were all greater than 0.7. The validity and reliability analysis results are summarized in Table 3.

**Table 3:** Validity and reliability test results.

Construct	Items	Factor loadings	Cronbach’s α	Construct	Items	Factor loadings	Cronbach’s α
Need for Cognition	NFC1	0.752	0.874	Collaborative Learning	CL1	0.839	0.907
	NFC2	0.808			CL2	0.758	
	NFC3	0.747			CL3	0.820	
	NFC4	0.761			CL4	0.892	
	NFC5	0.792			CL5	0.823	
	NFC6	0.717			CL6	0.824	
Self-Efficacy	SE1	0.712	0.861	Satisfaction	SAT1	0.752	0.934
	SE2	0.841			SAT2	0.800	
	SE3	0.815			SAT3	0.754	
	SE4	0.767			SAT4	0.710	
	SE5	0.722			SAT5	0.797	
	SE6	0.746			SAT6	0.805	
					SAT7	0.782	
					SAT8	0.825	
					SAT9	0.740	
					SAT10	0.795	
					SAT11	0.778	

#### Path Analysis

**Figure 3:**
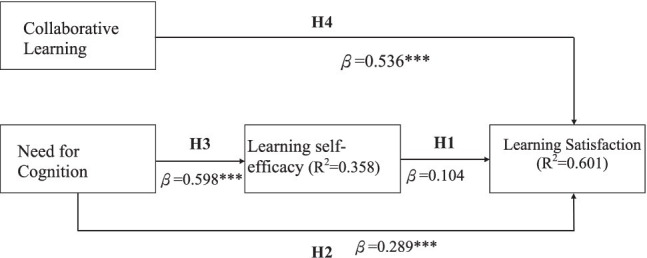
Path analysis results. ***p<0.05

In the structural model, the results were obtained by applying path analysis (as shown in Figure 3). In the structural equation model with cognitive needs, learning self-efficacy, and collaborative learning as the independent variables and learning satisfaction as the dependent variable, the adjusted R^2^ is 0.594; the model has a certain reference level. In the structural equation model with cognitive needs as the independent variable and learning self-efficacy as the dependent variable, the adjusted R^2^ is 0.354, and the explanatory power of the model reached a significant level.

In addition, the β coefficient of the path of learning self-efficacy to learning satisfaction is 0.104, and the *p* value is 0.117; the β coefficient of cognitive needs to learning satisfaction is 0.289, and the p value is 0.000; the β value of collaborative learning degree is 0.536, and the p value is 0.000. In addition, the β coefficient of the path of cognitive needs to learning self-efficacy is 0.598, and the p value is 0.000.

### Qualitative Research

#### Design of Interview Outline

Due to the finding in the quantitative research results that the level of collaborative learning has a very critical impact on learning satisfaction, more in-depth research will be conducted on collaborative learning after the quantitative research.

Since the interview was conducted in May, there had been a period of time since the end of class last semester. Therefore, in addition to referring to the items in the questionnaire that involve collaborative learning (Kitchen and Mcdougall, 1999; Driver, 2002; So and Brush, 2008), the questions of the interview outline were also designed to be combined with Tuckman’s five stages of group development (Tuckman and Jensen, 1977), and the questions were presented in a chronological manner to prevent the interviewee’s course experience from being distorted by time factors, as shown in Table 4.

**Table 4:** Interview outline.

No.	Interview questions
1	How were the group members formed into groups at that time, and what kind of assistance do you think the teacher can offer to groups in class?
2	The daily schedule among team members may be different, however, when discussions are needed, how you deal with it, have you had any special experiences or feelings in the process? (for example, it takes too much time and is not efficient)
3	In the learning process, if the group members have different ideas or opinions, how would you resolve the conflicts among yourselves? Will you choose to actively communicate your ideas with team members or choose to be submissive? And do you think these interactions will affect the attitude of team members in participating in this course?
4	In the team, do you have clear roles for each other? What do you think of your role in the team?
5	Are there any special learning experiences in the process of studying with students from different backgrounds and grades? How do you think this team interaction process will help you in your future employment?
6	What are your opinions and suggestions on the cooperation of the team members of this course?

#### Results of the Interview

The interview time ended within 15min on average, and sometimes, the order and direction of questions were adjusted according to the interviewee’s responses. During the interview process, questions outside the interview outline were also asked to get more in-depth details. The interviews were recorded using audio recording. Before recording, relevant explanations on research ethics and privacy were be provided to inquire about the interviewee’s willingness to record.

After the interview, the interview records were sorted into verbatim drafts based on the recording content, and after the interview records were converted into verbatim manuscripts, the words were segmented for each verbatim manuscript. The meaningless auxiliary words were removed, and the units with clear semantic meaning and readability were retained. After that, the meaningful units were coded. There were six codes in total; for example (consult the teaching assistant, or check it online to see if you have found a solution), the code from left to right is the interviewee’s number, interview question number, and verbatim serial number.

Interviewees codes 01 and 02 are for Marketing Management; 03 and 04 for Knowledge Creation and RandD Management; 05 and 06 for Entrepreneurial Management; 07 and 08 for Fundraising Platforms; 09 and 10 for Introduction to Computer and Internet Security; and 11 and 12 for the Reading Industry and Cultural Communication. When the interviewee’s number is an odd number, it means that the respondent was sampled from the high subgroup (top 25%); when a respondent number is an even number, it means that the respondent was sampled from the low subgroup (bottom 25%).

The two codes of the interview questions were based on the interviewee’s response to the question number of the interview at that moment and are noted with 01~07. The two codes at the end of the number are the serial numbers of a single independent verbatim manuscript that were marked as meaningful sentence units.

Finally, after sorting out all the coded sentences in each verbatim manuscript, the study performed thematic classification to obtain the classification result. Six topics about collaborative learning, including team formation, discussion atmosphere, discussion efficiency, decision-making mode, cooperation mode, and cross-domain learning, were obtained. The following section will discuss in detail each topic.

#### Formation of the Team

When the curriculum design is carried out in a flipped way, if there is not enough planning before the course for students to understand each other’s expertise and motivation to execute the project, the team composition tends to be random and members tend to find people who they already know to work with. This leads to the cohesion of team members to assume certain risks when the team executes the project, which indirectly affects the degree of classroom engagement; for example, “We started with a very fragmented consensus, because we all have different levels of expectations or understanding of this team.”

However, before the course starts officially, it is necessary to arrange some courses that can help students understand each other’s expertise and motivation. This will reduce unnecessary risks and help students find the right group before the course and is a better way to build team consensus when motivation is the same. Especially, when the project of the course involves interdisciplinary learning, it is also beneficial for students to combine their respective expertise for collaborative learning. For example, “there are some occupations in the publishing industry, such as editor-in-chief, editor-in-charge, and editor-in-art. Then, because the teacher has made this part of the assignment, the team members have a clear sense of their responsibilities and position. I think this is very helpful for grouping.”

It is worth noting that the consensus within the team will change with the development of the team, and the team goals may be more focused due to the organizational changes in the team. “After we started to do something, I think things were more on track.” When members were more willing to participate in the learning task of the course, members would also be more likely to focus on the overall goal of the team.

#### Discussion Atmosphere

The discussion process of collaborative learning may also lead to conflicts, which will affect the degree of engagement of the members. When there is a conflict in the discussion process, the group with a higher willingness to invest tends to face it actively and is more willing to take the initiative to put forward its own opinions and communicate with the members of the group: “We were livelier when we had meetings. We had a lot of trash-talking, so everyone … everyone felt that there is no sense of distance. Thus, we just kept throwing out ideas like this.” The group with a lower level of involvement was more inclined to avoid expressing their true ideas: “The discussions in our group are not particularly enthusiastic. It is more like ‘business is business’, that is, finish what you are responsible for and then hand over the results. Then nobody will raise too many objections; however, I do not think this is a good thing.”

One thing that can be noticed from here is that when team members encounter conflicts during a discussion, that does not deteriorate team relationships. When team members are more willing to communicate, moderate conflicts are often a boost for the team to generate innovative ideas.

#### Discussion Efficiency

Discussions between groups can be divided into three: (1) online plus in-person discussion, (2) online discussion, and (3) in-person discussion. From the feedback of the interviewees, it can be found that the efficiency of the group that only has online discussions is low. Online discussions often rely on social software, such as Line to communicate in asynchronous text. This mode of discussion may be inefficient because of the time gap in information: “It may be that everyone discusses a question, but the time taken by each person to answer it is different, and sometimes, it could be a long time. That is, the group must wait for everyone to give feedback and may have to wait for a long time.” In addition, this type of discussion often leads to team members only focusing on completing their assigned learning tasks and not communicating ideas.

Compared with the purely online text discussion, the physical discussion can encourage students to exchange more ideas, but there are also problems of inefficiency, and the underlying cause is often too many ideas among members. Opinions cause the discussion topics to lose focus, which is different from online discussions because of delays in the transmission of information; for example, “I think our group is mostly discussing… There are real discussions, where everyone would throw ideas, but we tend to have no conclusion.”

The online plus physical discussion approach has the characteristic of balancing the lack of the above two approaches. Face-to-face discussions ensure that participants exchange ideas, while implementation details can be tracked through an online communication software. It must be noted that compared to the form of pure online communication, this method focuses on communication software to track implementation details, rather than communicating ideas: “You can complete your large framework in the classroom, and the rest are the details. We have created a similar group for the details. If you have a problem or if you have any ideas, you can just type them and drop them in the group first.”

#### Decision-Making Mode

When team members face making important decisions, they can do so in two ways: (1) by reaching a consensus within the team members or (2) by seeking external resources. Consensus reached by group members can be further divided into two types: group members with clear roles and decentralization. Based on the results of this research interview, the group to which the sampled interviewees belonged generally tended to make important decisions in a decentralized manner. Compared to the group with a clear leader making decisions, it seems impossible to clearly point out the advantages and disadvantages, but from the results of this study, it is found that the decision-making model of group members without clear roles allowed each group member’s opinions to be fully heard and ensured that each member could participate in collaborative learning and jointly take the risk of decision-making; for example, “We will first listen to everyone’s opinions, and then, if there are different opinions, we will, for example, have different people come up with different solutions, after which we will analyse each solution individually and discuss the current situation, see the advantages and disadvantages of each plan, and, finally, see what solution will be most suitable for us.”

When there is no consensus within the group or when team members’ knowledge is not yet sufficient to make decisions, students will seek external resources such as classroom teachers. In the context of flipped teaching, when students’ abilities are not enough to cope with the problem, the teacher’s timely initiative to provide assistance is a link that must be paid attention to in flipped teaching design, which helps students to have a clearer direction when analyzing problems; for example, “When our team members just could not make a decision, we would ask the opinions of others, such as the teaching assistants or teachers, and then reach a unified opinion.”

#### Cooperation Mode

The results of this part of the text analysis are directly related to the formation of the first part of the team. In the initial stage of team formation, if students fully understand the specific expertise of the project that needs to be implemented in the course tasks, they will look for team members with relevant expertise when forming the team. The division of roles will also be more efficient in collaborative learning; for example, “Those who have a professional background are really good at certain aspects of tasks, that is, when they are good in the field, they will be relatively helpful, and they can do better than someone who spent the same amount of time.”

In contrast, if the division of labor cannot rely on the distribution of expertise among students, it will lead to a decline in efficiency: “Sometimes, I feel the function was allocated a little bit. The function was not evenly distributed, and then it did not show us what the team should be doing clearly.”

#### Interdisciplinary Learning

We can find from the results of text analysis that when the learning task of collaborative learning needs to be executed across domains, it is helpful to make use of the students’ own expertise, thereby enhancing students’ creativity, empathy, and even promoting mutual learning.

This result is directly related to the cooperation model in point (5). When the project is based on the division of expertise among group members so that the students’ own expertise or professional knowledge can be used, it will help improve the level of collaborative learning: “In fact, he might have learned this expertise in the club or in a school department, but because of this course and that we got together, we all have something to offer to the group. I think this is very important.”

In this learning process, students with different backgrounds of expertise can learn from each other and even improve their ability to innovate: “If we have different backgrounds, we may have different ideas. We may see different levels and different aspects. After discussion or communication, I may be able to understand why the other people would think a certain way, I can understand more things, or why I have never thought about it from his perspective. I need these things instead.”

Of course, in the process of communication, students will also improve their empathy and the ability to step into someone’s shoes, because they see the differences between group members: “If you work with people with varied information backgrounds, the points of concern will be different.”

It is worth noting that the above-mentioned positive responses can be observed regardless of the student’s learning effectiveness.

## Conclusion

### Research Findings

From the survey-based regression model, it is found that the cognitive needs of students and the degree of collaborative learning are directly related to the learning satisfaction of flipped teaching. This is in line with the focus of this study: Can specific personality traits be used to predict individual differences in students’ responses to flipped teaching? The results of this study prove that students with high cognitive needs have a relatively high degree of investment in the learning context of flipped teaching. The explanation for this phenomenon is the fact that flipped teaching in curriculum design requires students to show higher-order learning skills, such as analysis, judgment, and creativity in Bloom Taxonomy. Cognitive needs are directly related to these abilities. The research results of Nair and Ramnarayan (2000) prove that people with higher cognitive needs are more likely to succeed in solving problems. While Day et al. (2007) explored the association between cognitive needs and learning complex skills, they also confirmed that groups with high cognitive needs are helpful for learning complex skills.

In addition, the peer learning elements that flipped teaching emphasizes (Abeysekera and Dawson, 2015) are described as follows. The level of student engagement in the degree of collaborative learning also has a significant impact on the learning satisfaction of flipped teaching. In the regression model, it can be found that the β value of 0.536 for the level of collaborative learning is much higher than the 0.289 for cognitive needs. A phenomenon can be found here that the key factor affecting students’ investment in flipped teaching is that the needs of groups are greater than those of individuals. In fact, this result is not difficult to understand. When flipped teaching requires a large number of team-based methods, the interaction between the subjects and their peers in the classroom will naturally affect the degree of students’ involvement in the classroom. A study on the impact of teamwork on individual engagement and performance in the workplace environment also puts forward a similar viewpoint (Stashevsky and Levy, 2014), which argued that the quality of teamwork is what affects an individual’s willingness to engage in work.

In the relationship between cognitive needs and learning self-efficacy, the β value is 0.598 and has a significant impact, which proves that students with high cognitive needs will also have a higher level of learning self-efficacy, in other words, higher self-confidence, which is in line with our common sense judgment: When faced with a problem that requires time to think, students who like to think often have more confidence in solving the problem than students who are unwilling to think.

It should be noted that the results obtained in this study are mainly focused on learning satisfaction rather than learning effectiveness; thus, the impact of flipped teaching on learning effectiveness is not included in the discussion.

Thus, the qualitative study based on deep interview is worth to demonstrate the collaborative learning process of students participating the flipped learning. The results showed six important issues in facilitating the collaborative learning: team formation, discussion atmosphere, discussion efficiency, decision-making mode, cooperation mode, and cross-domain learning. (1) Team formation is the first important step of collaborative learning. The ice breaking activities helping students understand each other are an important mechanism before the course formally begin. (2) The building of discussion atmosphere is the second step facilitating collaborative learning, especially when there is a conflict in the discussion process. When team members are more willing to communicate, moderate conflicts are often a boost for the team to generate innovative ideas. (3) The discussion efficiency can be enhanced by using in-person discussion, and online discussion is discouraged for effective group discussion. (4) Team members showed two ways to achieve consensus—centralized and decentralized decision-making mode. (5) As team members have different background, the division of roles will be more efficient in collaborative learning. (6) If the team members corporate with each other by respecting the expertise, the team work efficiency can be improved.

Prior studies on flipped learning mainly focused on language teaching (Lee and Wallace, 2018; Andujar et al., 2020). This study is one of the limited studies that addressed the flipped learning in a variety of different courses, covering a wider range of domain and student background. Further, Tomas et al. (2019) explore how a flipped classroom supported students’ engagement and learning. The survey results suggested that the mechanism to enhance learning should be designed according to students’ learning needs and their readiness for a flipped learning approach. The results correspond to current study that the collaborative learning, atmosphere, and learners’ personality are important facilitators for learning outcome in flipped classroom.

To summarize, this study examined the predicting factors of learning satisfaction in flipped learning by using questionnaire survey, and the result suggested that collaborative learning if one of the most important predictors of learning outcome. Thus, a follow-up deep interview was further conducted to explore the collaborative learning process in flipped learning and six different important factors in collaborative learning were explored.

### Research Contribution

The results of this study prove that the level of collaborative learning is important to the engagement of students in the classroom. When implementing flipped teaching, apart from paying attention to students’ individual differences, it is also necessary to think about how to build a better team learning environment. This provides a direction of thought for educators who want to promote flipped teaching in the future.

In addition, according to the results of interview, when the course is carried out in groups, the teacher can arrange engagement motivation that can promote students’ understanding of each other’s expertise and course tasks. This enables students to find suitable groups before the course to facilitate the subsequent integration of their respective expertise. It also helps students build consensus within the team, and it is easier to build consensus within the team as the course is in progress. It can prevent students who do not know each other at all during the course from forming a group of students who may be inconsistent with their own goals for the course tasks. Besides, when the group members are faced with conflicts of views, if they can maintain good communication, it will help improve the students’ participation in the classroom; on the contrary, in the face of conflict, if there is no timely communication and mutual discussion among team members, the degree of students’ engagement in the classroom will be affected to a certain extent. When the students in the team do not have enough professional knowledge to reach a consensus in the face of conflict decision-making, it is very important to provide appropriate resource assistance in the classroom, such as teachers and teaching assistants. When the group is composed of members with diverse backgrounds, it will help enhance creativity and empathy and also enable students to contribute their knowledge and learn from each other in the process.

### Research Limitations and Future Research Directions

The first research limitation of this study is the small sample size caused by inviting only the participants from one of the six courses that meet the criteria of flipped teaching and get the permission of the instructors. Although the sample size is small, both qualitative and quantitative studies were conducted to answer the research questions deeply and broadly. Further, the courses considered in this study are restricted mainly in college of management and thus can limit the domains to be applied based on the current findings. As courses given in different domains (i.e., management, medical, science, and liberal) may have very different characteristics, the flipped teaching methods will also be different. Thus, the future studies can be suggested to include more courses in different domains in order to compare the facilitating factors of flipped learning satisfaction and outcome.

## Data Availability Statement

The raw data supporting the conclusions of this article will be made available by the authors, without undue reservation.

## Ethics Statement

Ethical review and approval was not required for the study on human participants in accordance with the local legislation and institutional requirements. Written informed consent for participation was not required for this study in accordance with the national legislation and the institutional requirements.

## Author Contributions

FF-C: study conception and design. P-CS: data collection. P-CS and C-SW: analysis and interpretation of results. FF-C and C-SW: draft manuscript preparation. All authors reviewed the results and approved the final version of the manuscript.

## Funding

Ministry of Science and Technology in Taiwan for financially supporting this research under Contract No. MOST-110-2410-H-029-023.

## Conflict of Interest

The authors declare that the research was conducted in the absence of any commercial or financial relationships that could be construed as a potential conflict of interest.

## Publisher’s Note

All claims expressed in this article are solely those of the authors and do not necessarily represent those of their affiliated organizations, or those of the publisher, the editors and the reviewers. Any product that may be evaluated in this article, or claim that may be made by its manufacturer, is not guaranteed or endorsed by the publisher.
